# How Decisiveness, Self-Efficacy, Curiosity and Independent and Interdependent Self-Construals Are Related to Future Hopefulness among Senior Students

**DOI:** 10.3390/bs9120154

**Published:** 2019-12-14

**Authors:** Hakan Remzi Öztekin, Fatih Bayraktar

**Affiliations:** 1 Faculty of Communication and Media Studies, Eastern Mediterranean University, Ismet Inonu St, 99628 Famagusta, Turkey; 2 Department of Psychology, Eastern Mediterranean University, Ismet Inonu St, 99628 Famagusta, Turkey; fatih.bayraktar@emu.edu.tr

**Keywords:** future expectations, self-efficacy, decision making, curiosity, emerging adulthood, cultural affiliation

## Abstract

Hope is a future-oriented reasoning that influences psychological assets of individuals. A hopeful standing towards the future can positively influence individual well-being. Different standings in terms of hopefulness may create variations in psychological assets of people. In the current study, we examined the associations of decisiveness, self-efficacy, curiosity and self-construals with hopefulness. A total of 278 senior university students were recruited for the study from Eastern Mediterranean University in Famagusta, North Cyprus. Participants filled six questionnaires; the Beck Hopelessness Scale, the Independent and Interdependent Self-Construal Scale, Multi-Domain Decisiveness Scale, Curiosity and Exploration Inventory II, the Dispositional Hope Scale and the General Self-Efficacy Scale. The data was analyzed by hierarchical multiple regression analysis on SPSS 23 software program. The results indicated that self-efficacy and decisiveness significantly predicted hopefulness, while curiosity did not predict hopefulness and only independent self-construal had the predictive effect on hopefulness. Based on these findings, the emerging adulthood nature of the study sample was discussed, and further recommendations were presented.

## 1. Introduction

The human mind is capable of representing future events in various flexible ways, imagining different possible outcomes and behaving accordingly in response to those representations. Hope is a particularly interesting concept among future-oriented reasoning because it is a powerful asset in the face of a challenging environment [1]. Snyder and his colleagues [2] conceptualized “hope” as not only a passive emotional process which pops up at the most exhausted or vulnerable moments but a goal-seeking process that has been actively pursued, “learned way of thinking about oneself in relation to goals” [3]. According to Snyder, hope is a cognitive process, specifically a cognitive set that is based on a reciprocally derived sense of successful agency (goal-directed determination) and pathways (planning to meet goals) [2].

Snyder’s Theory of Hope suggest that prior to starting a goal-oriented action sequence, two types of cognition are taking place in the mind: *pathways thinking* and *agency thinking.* He quoted that hopeful thinking is the sum of the mental will power and way power that you have for your goals [3]. Willpower represents agency thinking—the confidence one has to engage and maintain a goal-specific action sequence. Agency cognition is the reasoning that people have about their capability to initiate and maintain actions on the ways to meet goals [4]. It has also been articulated as a concept of self-determination to meet goals of the past, present and future [2]. Way power represents pathways thinking, referring to the perceived ability to generate ways to achieve goals. Pathways thinking reflects individual capacity to generate cognitive pathways to goals [4], which is activated when constructing plans or achieving goals. Because some plans may not succeed and some plans may succeed, hopeful thinking refers to being engaged in many plans in order to deal with possible obstacles in goal achievement progress. This study used Snyder’s Theory of Hope as a theoretical framework due to its comprehensive, active and multi-domain cognitive approach to the hope.

According to Snyder [5], hope can be related with various psychological assets such as optimism, self-esteem or personality traits. However, this study will focus on the other assets of psychological characteristics as predictors of hopefulness—self-efficacy, curiosity, decision making and self-construals. The importance of investigating hopefulness for senior university students is due to the fact that a particular role transition occurs after graduation [6]. Changes in social roles as well as changes in location can create distress or adaptation issues [7]. Understanding the dynamic behind this process is valuable in increasing the psychological well-being of students.

### 1.1. Self-Efficacy and Hopefulness 

Self-efficacy is a self-evaluation mechanism, which includes the belief in one’s capabilities to produce designated levels of performance that exert influence over events that affect lives [8]. Self-efficacy determines how to think, feel and motivate one’s self and eventually behave. High self-efficacy indicates the perception of challenging tasks as opportunities to be mastered instead of issues that have to be avoided [9]. Such a manner of thinking emerges from the assurance of one’s own capability. High self-efficacy sets individual’s commitment and maintenance of necessary energies to accomplish a challenge, as well as stability of the progress toward the goal [9]. According to Bandura [9], an efficacious way of thinking helps individuals to expect hopeful future results. He further explained self-efficacy as an enhanced self-determination level and the ability to predict the commitment necessary to fulfill an accomplishment. Furthermore, positive predictions tend to be thought of in self-efficacious way [9]. A number of studies investigated the positive effect of self-efficacy over future goal orientation processes and future aspirations in relation with general hopefulness [10,11,12]. Findings suggested a positive effect of self-efficacy over future hopefulness. 

### 1.2. Curiosity and Hopefulness

Curiosity can be described as the willingness to know, to experience, to see or to understand that which motivates exploratory actions toward something novel [13]. It is an intrinsic desire to experience or find knowledge that enables individuals to actively search the world, explore their environment and acquire knowledge about unknown or uncertain things [14]. Maw and Maw [15] described curiosity as a desire to know a novel stimulus that engages people in information seeking. It is referred to with positive affectivity, initiating exploration, the promotion of knowledge gathering, inspiration for information search and competence [16]. There were indirect findings in the literature [17,18,19] regarding the positive correlation of curiosity and future hopefulness, along with other variables. The current study will attempt to direct that association. 

### 1.3. Decision Making and Hopefulness 

Harris [20] defined decision making as the process of identifying and selecting alternatives based on characteristics of the one who makes decisions. Decisions are not only generated by basing them on as many alternatives as possible but the notion of the highest probability of success, suitability for personal goals, values, lifestyle, etc., Sinangil [21] suggested that decision making is the process of choosing the most suitable option out of many available. Decision making also has a role to reduce uncertainty and doubt in the alternatives via information gathering, and this progress includes defining the objective, collecting relevant information, generating feasible options, making a final decision, implementing it and evaluating the outcomes [22]. Accordingly, Moser [23] studied decision making as choosing the most beneficial, optimal and logical option among all available alternatives. Hopfensitz and Winden [24] conducted an experimental study to investigate the factors that influence risk attitude and decision making. According to their findings, hope had a positive effect on risk attitude during the decision-making process for investment planning within the experiment’s settings. Another study on decision making and future hopefulness was conducted by Chew and Ho [25], and they found that hope was helpful to resolve future uncertainty during the decision-making process.

### 1.4. Self-Construals and Hopefulness 

Markus and Kitayama [26] developed a self-construal model to explore the nature of individual experience including cognition, emotion and motivation. Self can be construed, organized or conceptually represented in various ways. Construal of self in a social context is related to the implicit effect of culture that moderates individual reasoning and behaviors to progress accordingly to what should be done. According to this model, the content of self may radically vary by culture. Some cultures perceive the individual in a sense of inner attributions of that person (independent self-construal). In contrast other cultures perceive the individual in a sense of belongingness to the social context and significant others (interdependent self-construal). Interdependent self-construal shapes the expression of emotions and motivations by the consideration of others, or consideration of self in relation to others, while independent self-construal shapes emotions and motivations by consideration of self-centered interests and personal values rather than social context-related interests and values. Furthermore, there is a conceptual link between hopefulness and self-construals—that is, having independent or interdependent self-construal effects cognition. Self-construals are part of self-schemas that have influence over information processing, emotional regulation and perception of self and environment [26]. Snyder [2] also discussed hopeful thinking as a complex cognitive process. Having independent or interdependent construct of self and/or social network can have an association with future-oriented reasoning. In other words, perception of self and others may create variations in future-oriented expectations. This study will try to test this relationship.

### 1.5. Emerging Adulthood and Hopefulness 

The term emerging adulthood describes the age period from approximately 18 to 25 [27]. It draws the changes and role transitions between adolescence and adulthood. At that period of time, in most industrialized countries, emerging adults obtain education or training, which creates the foundation of their future life [28]. Personal relationships, future plans, financial preparations, career achievements, etc., are some of the developmental tasks during emerging adulthood [27]. Due to its higher opportunity nature and energetic biological foundation than adulthood [29], emerging adults view and maintain their personal future with high hopes [27]. Emerging adults have more choices than previous cohorts and these choices lead to greater opportunities and higher levels of future hopefulness [30]. Since there are still open doors for many future possibilities, optimistic and hopeful feelings toward the future is suitable for the emerging adulthood period [30]. Whether all university students can be counted as emerging adults or not, Cavanaugh [31] discussed that majority of university students that have taken traditional education within industrialized countries are in the emerging adulthood period of development. According to the National Center for Education Statistics of U.S [32], college students represent a major and distinct portion of the emerging adulthood population.

### 1.6. Current Study

Due to the changing and uncertain life conditions after graduation including changes in social roles and status [6], differentiations among senior university students are expected when it comes to future-oriented feelings in the last year of university life. Characteristics of the emerging adulthood period of development and the senior year of university experience have similar features in nature and are, therefore, considered as a discussion matter in the current study. Similar suggestions of self-efficacy and agency thinking in Snyder’s Theory of Hope [2] generated the first hypothesis of the study. In the same manner, structural similarities of the decision-making process and pathways thinking of Snyder’s Theory of Hope [2] generated a second hypothesis to explore. Furthermore, uncertainty of the life course after graduation was also considered to be related with curiosity, which refers to being tolerant to the uncertainty concept [33] and preferring new and uncertain experiences [34]. Therefore, this was considered to be worth exploring and generated the third hypothesis. In addition, cultural differentiation as a macro influence on hopefulness was explored by evaluating self-construals. Despite the study sample consisting of Turkish-speaking participants, and that the Turkish culture is known to have a more interdependent social context [35,36,37], the fourth hypothesis of the study stated that independent self-construal will predict hopefulness, due to its individual-promoting efficacious nature.

Based on those motives, hypothesis one was that the participants who have high levels of self-efficacy will have high levels of hopefulness, hypothesis two was that the participants who are more decisive will have high levels of hopefulness, hypothesis three was that the participants who have high levels of curiosity will have high levels of future hopefulness and hypothesis four was that the participants who have an independent view of self will have more future hopefulness than participants who have an interdependent view of self.

## 2. Materials and Methods

### 2.1. Participants

A total of 278 senior university students of a public university in Famagusta, North Cyprus, were recruited for the study. All participants were Turkish-speaking students and 89.7% of participants were between 20 and 25 years of age (M = 22.13, SD = 1.92), 3.7% of participants were below the age of 20 and 6.6% of participants were above the age of 25. One hundred and one participants (36.3%) were male, 150 participants (54%) were female, and 27 participants (9.7%) did not specify their gender. Eighty one psychology students (29.1%), 85 law students (30.6%), 8 fine arts students (2.9%), 18 psychological counselling and guidance students (6.5%), 29 business and economics students (10.4%) and 6 educational sciences students (2.2%) specified their department and 51 (18.3%) participants did not specify their department.

### 2.2. Measures

#### 2.2.1. Self-Construals 

The Independent and Interdependent Self-Construal Scale was developed by Lu and Gilmour [38] to measure independent and interdependent self-construals. This is a 7-point Likert scale, with 42 items. Lu and Gilmour found that the Cronbach alpha of independent and interdependent self-construals was 0.86 and 0.89, respectively. Bayraktar [39] translated and adapted this scale into the Turkish language. According to their findings, confirmatory factor analysis showed that the two-factor model had an adequate fit to the data, which means that independent and interdependent self- subscales could be used in Turkish-speaking populations (the Cronbach alpha for the independent subscale was 0.89 and the interdependent subscale was 0.87). In the current study, the Cronbach alpha for the independent self-construal subscale and the interdependent self-construal subscale was 0.83 and 0.89, respectively, and the overall scale internal consistency coefficient was 0.87. Higher points in a subscale mean more tendency to have this specific subscale’s self-construal.

#### 2.2.2. Decision Making 

The Multi-Domain Decisiveness Scale was developed by Haraburda [40] to assess the general and personal decision-making ability of individuals. This is a 6-point Likert scale questionnaire. Sarı [41] adapted this scale for the Turkish-speaking population. According to his findings, the internal consistency coefficient was 0.86 for the overall scale, 0.64 for the general decisiveness subscale, 0.62 for the conflict solving subscale, 0.73 for the certainty choice of social relations subscale and 0.56 for the easiness choice of the social relations subscale. In the current study, the overall score of the scale is decided to evaluate general decisiveness because the measurement validity of the scale is consistent with the research hypothesis. The overall Cronbach alpha was 0.80 for the study sample. Higher points indicate higher decisiveness.

#### 2.2.3. Curiosity 

Curiosity and Exploration Inventory II was developed by Kashdan et al. [42] and is used to measure the curiosity levels of participants. This is a 5-point Likert scale, with 10 items. Kashdan and his colleagues conducted three different psychometric studies while developing the scale and the Cronbach alpha ranged between 0.75 and 0.86 in those studies. This scale has two subscales—stretching (motivation to seek out new knowledge and experience) and embracing (willingness to embrace the novelty and uncertainty). Acun, Kapıkıran and Kabasakal [43] translated and adapted the scale into the Turkish language. Their analysis showed that the two subscales were highly related (r = 0.85), and that the Cronbach alpha of the overall scale was 0.81. The internal consistency was 0.81 and 0.68 for stretching subscale and embracing subscale, respectively. In the current study, we decided to use the overall score of the scale for evaluation due to the close nature of the subscales and general validity of the scale on curiosity. The overall Cronbach alpha of the scale was 0.70. There were no reverse items in the scale and higher points indicate higher curiosity.

#### 2.2.4. Hopefulness 

The Dispositional Hope Scale was developed by Snyder et al. [2] to assess the hopefulness levels of individuals. This is an 8-point Likert scale, with 12 items. The scale has two subscales—alternative ways of thinking (represents pathways thinking) and actual thinking (represents agency thinking). According to the psychometric study of Snyder and his colleagues, the Cronbach alpha for the alternative ways of thinking subscale was between 0.64 and 0.80; for the actual thinking subscale, it was between 0.71 and 0.76; for the overall scale, it was between 0.74 and 0.84. Tarhan and Balcalı [44] translated and adapted this scale for the Turkish-speaking population. According to their findings, the Cronbach alpha for the overall scale was 0.83. We decided to use the overall score of the scale as a measurement due to its general validity to measure hopefulness. In our study, the Cronbach alpha of the overall scale was 0.85. There was no reverse item in the scale, and the 3rd, 5th, 7th and 11th items were fillers. Higher scores indicate higher hopefulness.

#### 2.2.5. Self-Efficacy 

The General Self-Efficacy Scale was developed by Schwarzer and Jerussalem [45] to assess the self-efficacy levels of individuals. This is a 5-point Likert scale, with 10 items. Reliability studies of the scale were conducted in three different countries (Germany, Spain and China) and the Cronbach alpha of the scale was between 0.78 and 0.91 [46]. The scale was translated and adapted into the Turkish language by Aypay [47]. According to the results of this study, the Cronbach alpha of the scale was 0.83. There was no reverse item in the scale and higher points indicate higher self-efficacy. In the current study, the Cronbach alpha of the scale was 0.86.

### 2.3. Procedure

After obtaining approval from the Research Ethics Committee (Approval code: ETK00-2018-0108), classes of senior-year university students were set. Data was collected in classroom settings during class times. Both the researcher and the lecturer of the class were present during data collection. First, students were informed about the study and voluntary students signed the informed consent. Students took the scales and filled individually them in the classroom. Participants were also asked to write down their demographic information on the first page of the scales. After finishing the surveys, students took a debriefing form. Scales were filled in a single session and each session took approximately 20 min. In the current study, SPSS 23 software was used for the analyses.

### 2.4. Statistical Analysis

The Pearson Correlation Coefficient was used to determine the relationship among the variables. Then, a hierarchical multiple regression analysis was conducted to determine the predictor roles of independent variables on hopefulness.

## 3. Results

### 3.1. Correlations among Continuous Variables

As seen in Table 1, there were positive correlations between hopefulness and decisiveness, self-efficacy, curiosity, and self-construals. Self-efficacy had the strongest correlation coefficient with hopefulness.

### 3.2. Hierarchical Multiple Regression Analysis Findings for Variables Predicting Hopefulness

In the present study, hierarchical multiple regression analysis was conducted to examine the predictor role of age, curiosity, decisiveness, self-efficacy and self-construal types on hopefulness (See Table 2). Preliminary analyses were conducted to ensure no violations of the assumptions of normality, linearity, multicollinearity and homoscedasticity. Examination of the data indicated significance and assumptions were met for VIF (variance inflation factor) and tolerance. The highest VIF of all variables was 1.527 for self-efficacy. The lowest tolerance to hopefulness was 0.655 for self-efficacy.

In the first step of regression analysis, age was entered as the control variable. The model was insignificant (F (1241) = 1.239, *p* = 0.267), and explained 0.005% of the variation in hopefulness. Individual-related variables, curiosity, self-efficacy and decisiveness, were entered in the second step while controlling age (F (4241) = 74.794, *p* < 0.001). The second model explained 55.8% of the variation in hopefulness with decisiveness (β = 0.181, *p* = 0.001) and self-efficacy (β = 0.628, *p* < 0.001). Self-construals were introduced in the last step as culture-related variables (F (6,241) = 55.285, *p* < 0.001). The third model explained 58.5% of the variation in hopefulness with independent self-construal (β = 0.170, *p* = 0.001), self-efficacy (β = 0.576, *p* < 0.001), decisiveness (β = 0.175, *p* < 0.001) and age (β = −0.084, *p* = 0.051). Curiosity and interdependent self-construal were not significant predictors in the third step.

## 4. Discussion

The present study explored the effects of self-efficacy, decisiveness, curiosity and independent and interdependent self-construals on the hopefulness of senior-year university students. It was found that self-efficacy and decisiveness significantly predicted the hopefulness of senior students. However, curiosity was found to have no prediction role in hopefulness. Furthermore, only independent self-construal predicted hopefulness. Age did not have a prediction role in hopefulness.

Findings of the study supported the first hypothesis that participants who have high levels of self-efficacy will have high levels of future hopefulness. According to the results, hopefulness was significantly predicted by self-efficacy, and this finding is supported by the literature [10,11,12]. Such a finding can also be linked to the literature on hopefulness in two ways. Firstly, Bandura stated the importance of self-efficacy over future-oriented expectations in multiple occasions [8,9]. The current study findings were parallel with Bandura’s self-efficacy model in a way that the suggestion of future-oriented expectations together with high self-efficacy enable individuals to think hopefully about future. The second link between high self-efficacy and hopefulness could be related to Snyder’s Theory of Hope itself. The parallel suggestions of Bandura’s self-efficacy model and agency thinking by Snyder may highlight the compactness of the two models. Snyder’s agency thinking and Bandura’s self-efficacy definitions have common aspects. Snyder and his colleagues [2] articulated agency thinking as a reasoning that people have about their capability and efficacy to initiate and maintain actions on selected pathways to meet desired goals. Similarly, Bandura [8] described self-efficacy as the belief in one’s capabilities to execute the necessary actions that influence events that affect one’s life. Clearly, Snyder’s agency thinking refers to efficacious reasoning on future. Such mutuality has already been recognized by Snyder as well [2], as he stated that agency thinking represents efficacious thinking. However, according to his model, both agency thinking and pathways thinking are necessary for hopefulness. Agency thinking and pathways thinking are indistinguishable in maintaining hopeful thinking—inefficiency in one component will eventually lead to dysfunction in the other component and eventually lead to issues in hopefulness. Despite the binary dynamic of Snyder’s model, the current study findings may indicate that efficiency in self-efficacy can escalate hopeful thinking due to its natural link with agency thinking. Further research is recommended to investigate how much of a role agency thinking plays in hopeful thinking while controlling pathways thinking. Extending research to the investigation of the dynamics of hopeful thinking may enlighten our understanding of the role of self-efficacy in hopefulness.

The developmental stage of current study sample is also worth noting to indicate the importance of self-efficacy on future hopefulness. According to Arnett [30], emerging adults tend to be hopeful and efficacious due to the energetic nature of this period and future possibilities. The opportunistic nature of the emerging adulthood period of development and the characteristically optimistic features of emerging adults are important factors for future hopefulness. Findings of the current study support the link between the efficacious characteristics of emerging adult participants and its predictive association with hopefulness.

Findings of the study also support the second hypothesis—participants who are more decisive have high levels of future hopefulness. Decisiveness predicted hopefulness significantly in the current study and other studies within the literature supported our finding [24,25]. This finding may be linked with the pathways thinking component of Snyder’s Theory of Hope due to particular similarities. Snyder and his colleagues [3] described pathways thinking as the perceived ability to generate ways to achieve goals. Similarly, the literature defined the decision-making process as generating alternative ways to achieve the goal (pathways to the outcome) and choosing the most appropriate and optimal way to reach the desired goal [20,21,22,23]. An important asset of the decision-making process is generating suitable alternative ways to achieve the desired outcome and this aspect is similar to Snyder’s pathways thinking concept. Furthermore, Snyder [3] mentioned that facing obstacles and challenges trigger hopeful thinking. However, engaging in flexible and various plans to deal with negative issues during goal achievement and being able to initiate new plans on the way to goals are important assets for a hopeful thinker [3]. That ability to generate new plans in a goal-oriented approach is referred as decent decision-making ability [22].

Positive prediction of decision-making ability in hopefulness also fits in with the definitions of emerging adulthood. The literature suggests that emerging adults are more prone to being optimistic and hopeful about the future due to having opportunities, chances and more decisions for the future. Findings are linked with the nature of the future in emerging adulthood [27,29,30] and highlight being hopeful during decision making about future. 

Findings of the current study also support the fourth hypothesis—participants who have an independent view of self will have high levels of future hopefulness. Independent self-construal had a predictive role in hopefulness according to our findings. This result is discussed by Bernardo’s work found in the hope literature [48]. He extended the hope theory of Snyder and added two new dimensions, internal locus of hope and external locus of hope. According to him, the internal locus of hope indicates hopeful thinking that has been construed based on the person-centered attributes. In contrast, the external locus of hope indicates hopeful thinking that relies on significant others. Du and King [49] also demonstrated that an independent view of self tends to possess an internal locus of hope, while an interdependent view of self tends to enjoy an external locus of hope. This extension is particularly important because of Bernardo’s discussion in the hope literature. According to Bernardo [48], the dimensions of hope require further research to understand them comprehensively. Hope is not only a personality trait or a learned view of the future—external agents (i.e., family, friends) or spiritual beliefs can play a role in hopeful thinking. Based on his suggestions, results of the current study need to be further studied to examine an interdependent view of self and its association with hopefulness more deeply. This limitation of the study indicates that future research must be conducted to investigate the association between hopefulness and both self-construals.

Results of the current study can be useful to individual well-being services in universities. Most of the universities around the world have psychological counselling centers that provide psychological support to students. Having an understanding that self-efficacy and decisiveness increases the future hopefulness of university students may increase the efficiency of professionals in those centers to help students. In the current study, the strongest association of hopefulness was found with self-efficacy. This finding can be linked to the lecturer’s attitudes during university education. Being a positive, helpful and understanding lecturer can help students to develop healthy self-efficacy and lead to better future-oriented standing. Lecturers in universities should consider how university education can be challenging and how it can affect student’s self-efficacy and must behave accordingly. Furthermore, parents, as role models, should promote self-efficacy development early in their child’s life. This may ensure healthy and functional future hopefulness for children at their emerging adulthood stage of development. Further, similar studies should be conducted in other countries to determine whether there is a difference based on socio-geographic factors in terms of hopefulness.

The current study had particular methodological limitations. The data collection procedure was conducted inside a social environment—classroom settings. The presence of others can interfere with an individual’s way of thinking including self-efficacy [50], and classmates may create an in-group perception that eventually may lead to a confounding effect during data collection. All data were collected via self-report questionnaires. A qualitative methodology could expand our understanding of hopefulness and its correlations further. Moreover, different departments of universities may have different future pathways due to the varied nature of jobs and opportunities. Departmental differences may carry variations in the hopefulness of university students. However, the current study did not investigate this adequately. Future research could open new research topics on departments and their perceived future expectations. Such research may provide re-evaluations of occupational policies for organizations and governments. Hopefulness as a positive psychology topic is particularly worth studying for the sake of individual well-being during preparation for the future. As Jean-Baptiste Andre Godin once said, “the quality of our expectations determines the quality of our actions”.

## Figures and Tables

**Table 1:** Correlations among Hopefulness, Age, Decisiveness, Self-Efficacy, Curiosity and Self-Construals.

Variables	1	2	3	4	5	6	7
1. Hopefulness	-						
2. Age	0.072	-					
3. Decisiveness	0.451 **	0.150 *	-				
4. Self-Efficacy	0.721 **	0.191 *	0.420 **	-			
5. Curiosity	0.369 **	0.088	0.235 **	0.403 **	-		
6. Independent Self-Construal	0.404 **	0.059	0.203 *	0.324 **	0.218 **	-	
7. Interdependent Self-Construal	0.136 *	0.115 *	−0.076	0.178 *	0.075	0.086	-

* *p* < 0.05; ** *p* < 0.0001.

**Table 2:** Hierarchical Multiple Regression Analysis Findings for Variables Predicting Hopefulness.

		Model 1			Model 2			Model 3	
Variables	β	SE B	t	β	SE B	t	β	SE B	t
Age	0.072	0.032	1.113	−0.082	0.022	−1.867	−0.084	0.021	−0.1.958 *
Decisiveness				0.181	0.072	3.776 **	0.175	0.071	3.690 **
Self-Efficacy				0.628	0.088	12.288 **	0.576	0.089	11.100 **
Curiosity				0.080	0.089	1.699	0.063	0.087	0.1.358
Independent Self-Construal							0.170	0.073	3.803 **
Interdependent Self-Construal							0.037	0.045	0.850
R^2^		0.005			0.558 **			0.585 **	

* *p* < 0.05; ** *p* < 0.0001. β: standardized regression coefficients; SE B: unstandardized beta; t: *t* test statistics.
